# Functionality and Palatability of Yogurt Produced Using Beetroot Pomace Flour Granulated with Lactic Acid Bacteria

**DOI:** 10.3390/foods10081696

**Published:** 2021-07-22

**Authors:** Marina Jovanović, Snežana Zlatanović, Darko Micić, Dragan Bacić, Dragana Mitić-Ćulafić, Mihal Đuriš, Stanislava Gorjanović

**Affiliations:** 1 Institute of General and Physical Chemistry, Studentski trg 12/V, 11158 Belgrade, Serbia; snezana.zlatanovic@gmail.com (S.Z.); micic83@gmail.com (D.M.); stasago@yahoo.co.uk (S.G.); 2 Faculty of Veterinary Medicine, University of Belgrade, Bulevar oslobođenja 18, 11000 Belgrade, Serbia; bacicd@vet.bg.ac.rs; 3 Faculty of Biology, University of Belgrade, Studentski trg 16, 11158 Belgrade, Serbia; mdragana@bio.bg.ac.rs; 4 Institute of Chemistry, Technology and Metallurgy—National Institute of The Republic of Serbia, University of Belgrade, Njegoševa 12, 11000 Belgrade, Serbia; mihal.djuris@ihtm.bg.ac.rs

**Keywords:** vegetable pomace, dairy beverage, fluidized bed, heat-sensitive compounds, functional food, palatability, by-products, *Canis familiaris*, DIY formula, sustainability

## Abstract

Following the idea of sustainability in food production, a yogurt premix based on beetroot (*Beta vulgaris*) pomace flour (BPF) was developed. BPF was granulated with lactose solution containing lactic acid bacteria (LAB) by a fluidized bed. Particle size increased ~30%. A decrease in Carr Index from 21.5 to 14.98 and Hausner ratio from 1.27 to 1.18 confirmed improved flowability of granulated BPF, whereas a decrease in water activity implied better storability. Yogurts were produced weekly from neat starters and granulated BPF (3% *w*/*w*) that were stored for up to one month (4 °C). High viability of *Streptococcus thermophilus* was observed. Less pronounced syneresis, higher inhibition of colon cancer cell viability (13.0–24.5%), and anti-*Escherichia* activity were ascribed to BPF yogurts or their supernatants (i.e., extracted whey). Acceptable palatability for humans and dogs was demonstrated. A survey revealed positive consumers’ attitudes toward the granulated BPF as a premix for yogurts amended to humans and dogs. For the first time, BPF granulated with LAB was used as a premix for a fermented beverage. An initial step in the conceptualization of a novel DIY (do it yourself) formula for obtaining a fresh yogurt fortified with natural dietary fiber and antioxidants has been accomplished.

## 1. Introduction

The growing awareness of nutrition and the environment has directed the flow of current research toward the usability of agri-food by-products. The long-term focus on this approach is supported by the objectives of the Food and Agriculture Organization’s (FAO) Sustainable Development Goals, Farm to Fork Strategy 2020, the FOOD Strategy 2030, and Serbian and European Bio-Economy Strategy [1,2,3]. The root vegetable *Beta vulgaris* L. (fam. Chenopodiaceae), commonly known as beetroot, is globally consumed as part of the normal diet and used to fabricate a natural food coloring agent known as E162 and ready-to-drink nonalcoholic beverages. Large quantities of beetroot pomace (BP) are available as a by-product [4,5].

The restoration of minimally processed BP back to the food chain can enhance the nutritional characteristics of processed food. The considerable potential of BP utilizations lays in the high dietary fiber (DF) content [5,6], a moderate caloric value, and the significant amount of antioxidants (AOs), such as nitrogenous pigments (betacyanins and betaxanthins) and polyphenolics (ferulic, protocatechuic, gallic and caffeic acid, catechin, and myricetin) [7,8,9]. To date, beetroot has been used as an ingredient in fermented juice, cereal bars, beetroot-enriched bread [10], chips [11], vegetable-based smoothies [12], and yogurts [13]. The majority of listed products contain lactic acid bacteria (LAB) [10,11,13]. However, none of them were obtained using BP with immobilized LAB. The immobilization can increase the viability of LAB by promoting the adherent properties of microorganisms and mimicking cell growth within natural structures. Known as good immobilization materials, DF, fruit, and vegetable powders were already used as immobilization support for yogurt starter cultures [14].

Fluidized bed granulation is often a method of choice for drying heat-sensitive compounds, including microbial cultures. Advantages, such as good temperature control, equal temperature distribution, high drying capacity, short drying time, low maintenance costs, and large-scale production, are noticeable [15,16,17,18]. To minimize the damage caused by drying and to achieve increased bacterial viability in storage time, fluidized bed immobilization of LAB is mainly performed in the presence of protective carbohydrates [15]. Lactose, for instance, interacts with the polar section of membrane lipids, providing protection from cell degradation during drying and storage [19,20].

Yogurt, a worldwide consumed beverage with beneficial effects on the immune system, gastrointestinal health, and a reduction of specific cancer risk [21,22], is a fermented dairy product that is acidified and coagulates owing to the activity of *Lactobacillus delbrueckii* subsp. *bulgaricus* and *Streptococcus thermophilus* [23]. It is highly recommended for immunocompromised populations and those suffering from lactose intolerance [21]. Various probiotics strains could also be added in the dairy products intended for humans and their companion animals [24]. LAB enables the proteolytic release of peptides with anticarcinogenic potential from yogurt proteins. Various food-derived peptides also exhibit cytotoxic activities against malignant cells, usually by disrupting cell membranes [22]. Fermentation improves the nutritional value, palatability, and preservative and medicinal properties [25]. Functional features can be further improved by adding fruits and vegetable pomace [13,26].

Modern consumers favor green consumerism, take special notice of their food choices, and raise the question of the eating habits of their companion animals, particularly dogs (*Canis familiaris* L.) [6]. Functional foods intended for dog nutrition have gained considerable popularity [25,27]. Moreover, pet parents prefer food that resembles their meals [6]. The concept of sharing food with pets, such as commercially available snack bars (Yaff BAR, Mudd & Wyeth, South Hero, VT, USA), has received increasing attention. Functional human or commercial pet food contains beet as a source of DF and AOs [6]. Therefore, a fermented dairy beverage with BP could have a positive tag appeal for pet owners and the wellness generation.

In this study, the main purpose was to granulate BP flour (BPF) with LAB and evaluate the applicability of the obtained granulate as a yogurt premix. Wet granulation of BPF in a fluidized bed was employed to improve the physical properties of BPF and to utilize it as LAB support. The physical characteristics of BPF before and after granulation were compared. For one month, neat and immobilized starters were stored and used weekly to produce plain and BPF yogurts. The functionality and palatability of fresh yogurts were investigated. A decrease in pH during fermentation and occurrence of syneresis was followed. To highlight functionality, plain and BPF yogurts were analyzed from the microbiological point of view. LAB viability and anti-*Escherichia* activity were estimated. Further, the cytotoxic effect of yogurts’ supernatants (i.e., extracted whey) on the human colon cancer cell line (HCT116) was surveyed. An in vivo toxicity test was performed to confirm the novel product safety before palatability surveying by both humans and dog trials. Finally, to gain insight into the public attitudes toward the BPF premix for yogurts amended to humans and dogs, a survey was conducted. This study represents a step toward the conceptualization of an affordable DIY (do it yourself) formula for obtaining a fresh yogurt fortified with natural DF and AOs, suitable for both human and dog consumption.

## 2. Materials and Methods

### 2.1. Materials

Pasteurized cow milk (Dairy plant “Zapis Tare”, Serbia) and red beetroot (Detroit variety) were purchased from the local markets (Belgrade, Serbia). Commercial raspberry yogurt for dogs (“Creamy Timmy “, Creamy Jimmy) was purchased from the local pet shop (Belgrade, Serbia).

Lyophilized starter culture YC-X11, containing *Lactobacillus delbrueckii* subsp. *bulgaricus* and *Streptococcus thermophilus* was obtained from Chr. Hansen (Hørsholm, Denmark). Bacterial strain *Escherichia coli* ATCC 8739 was obtained from the Department of Microbiology, University of Belgrade—Faculty of Biology, Serbia.

Human colon cancer cell line HCT116 (ATCC CCL-247) was obtained from the Oncology Institute of Vojvodina, Serbia.

M17 agar, De Man-Rogosa-Sharpe agar (MRS), Müller Hinton broth (MHB), and Mac Conkey Agar were obtained from HiMedia (Mumbai, India). Dulbecco’s modified Eagle’s medium (DMEM), penicillin–streptomycin mixtures, phosphate buffered saline (PBS), trypsin from the porcine pancreas, and 3-(4,5-dimethylthiazol-2-yl)-2,5-diphenyltetrazolium bromide (MTT) were purchased from Sigma-Aldrich (Steinheim, Germany).

### 2.2. Beetroot Pomace Flour Preparation and Characterization

The beetroot pomace (BP) collected immediately after squeezing thoroughly washed beets (Bosch GmbH, Renningen, Germany) was subjected to dehydration, in the laboratory dehydrator (Excalibur, model 3926 TB, Sacramento, CA, USA) for 480 min at ≤55 °C, and to subsequent grinding (>200 µm, determined by sieving). The proximate composition of BPF was determined. The moisture level was determined at 105 ± 5 °C in a thermostatically controlled dry oven until a constant weight was reached. The proximate composition of BPF, including protein (Nx5.30) (Official Method No. 950.36), fat (Official Method No. 935.38), crude fiber (Official Method No. 962.09), and ash (Official Method No. 930.22), was determined by standard AOAC methods [28,29]. The carbohydrate content was calculated by subtracting the sum of moisture, ash, crude fiber, fat, and protein from 100%.

### 2.3. Batch Granulation of BPF by Fluidized Bed at Pilot-Scale Level

The process of wet granulation in a fluidized bed (pilot-scale device constructed and built within the scope of Innovation Project IP 16-10 2018 funded by the Ministry of Education, Science and Technological Development of the Republic of Serbia) was performed to obtain granulated BPF. BPF granulation implied immobilization of a yogurt starter culture (YC-X11) onto BPF. BPF (300 g) was sterilized in the preheated column (78 °C) of a fluidized bed dryer for 10 min. The temperature in the column was reduced to 48 °C. The BPF was granulated via top spraying of aqueous lactose solution (5% *w*/*v*; 150 mL), mixed with a bacterial starter culture (2 g). The pressure through a two-fluid nozzle was 1.8 bar. The mean fluid flow rate and air flow rate were 5 mL/min and 35 m^3^/h, respectively. The granulated BPF was dried by fluidization at 40 °C for 15 min. To prevent disintegration of the formed granules due to collision and attrition, the air flow rate in the drying phase was reduced to 30 m^3^/h. After the drying phase, the samples were cooled to room temperature, transferred into food-grade sterile polypropylene (PP) zip lock bags, and maintained at 4 °C for a one-month period.

### 2.4. Determination of Physical and Technological Properties of BPF before and after Granulation

Physical and technological properties of BPF before and after granulation, including bulk and tap density, flowability, particle size distribution, and water activity (aw), were compared.

#### 2.4.1. The Flowability

Bulk density measurement was performed in a glass cylinder of 73.95 mL in volume (23 mm in diameter and 178 mm in height). The tapped density was obtained in the same cylinder by mechanically tapping until the flour surface reached the maximum packing condition (300–400 taps). The measurements of bulk and tapped densities were repeated three times, and the mean value was used to calculate the Carr Compressibility Index (C_I_) and Hausner ratio (H) that defines the flowability.

(1) $$C_{I}=100\times \frac{\rho_{\mathrm{tapped}}-\rho_{\mathrm{bulk}}}{\rho_{\mathrm{tapped}}}$$

(2) $$H=\frac{\rho_{\mathrm{tapped}}}{\rho_{\mathrm{bulk}}}$$

where ρ_bulk_ and ρ_tapped_ are bulk and tapped density of material in g/mL, respectively.

#### 2.4.2. Particle Size Distribution

The particle size distribution was determined by analyzing a scanned sample image (between 1000–2000 particles) using 2D image analysis software ImageJ (Developed by University of Wisconsin-Madison, Madison, WI, USA, under Public Domain, BSD-2 license). Images were captured by a 2D scanner Hp Scanjet 300 and stored at the high resolution of 4800 dpi, as described [30]. Since the particles are irregular in shape, the size of the particles was expressed as the projected area diameter d_A_.

(3) $$d_{A}=\sqrt{\frac{4\times A}{\pi }}$$

where A is the surface area of the projection of the particle; a sum of pixels areas in calibrated units (e.g., μm^2^).

#### 2.4.3. Water Activity

For one month, aw of non-granulated and granulated BPF was determined weekly by the aw meter Novasina LabSwift Bench-model Water Activity Meter (Neutec Group Inc., Farmingdale, NY, USA) at 25 ± 2 °C. For each sample, three independent experiments in triplicates were performed.

### 2.5. Evaluation of Granulated BPF Toxicity

Acute toxicity was examined to confirm the safety of granulated BPF. The care and treatment of laboratory animals were performed according to the regulations and standards of the national (Serbian) Law on the Experimental Animal Treatment and European Directive 2010/63/EU (European Convention for the Protection of Vertebrate Animals used for Experimental and other Scientific Purposes). Four-week-old pathogen-free Han: NMRI mice (*n* = 5), with initial body weights of 18–22 g, were housed in standard cages in a room with a 12 h light-dark cycle at a temperature of 22 ± 3 °C. Mice were fed with granulated BPF (50 mg) mixed with water once using a gastric probe. The observation period was 72 h.

### 2.6. Application of Granulated BPF as a Premix in Yogurt Production

#### 2.6.1. Production of Yogurt at Laboratory Scale Level

Neat starter cultures and a BPF premix (i.e., granulated BPF) stored for 0, 7, 14, 21, and 28 days were used to prepare a control yogurt and BPF yogurt, respectively. Yogurts were prepared once a week from BPF premix in order to determine if there was a difference in fermentation rate. The cow milk (2.8% milk fat) was heat-treated at 85 °C for 10 min, cooled down to 43 °C, and poured into glass containers (100 mL) and neat (0.02 g) starter culture or BPF premix (3% and 9% *w*/*w*) were added. After thorough mixing, the samples were subjected to fermentation at 43 °C until a pH < 4.6 was reached. The pH values were determined using a pH meter with a gel-filled electrode (WTW™ SenTix™ 41 pH, California, CA, USA). Obtained yogurts were stirred and stabilized by cooling (4 °C for 24 h). Freshly prepared yogurts with 3% *w*/*w* BPF premix were used in all further testing except in the cytotoxicity test. Yogurt containing 9% BPF premix was further processed and used in the cytotoxicity assay. All samples were made in triplicate, and the samples preparation was repeated three times.

#### 2.6.2. Syneresis of Yogurt Samples

The samples (2 × 25 g) from each batch of yogurt were weighed in centrifuge tubes, centrifuged at 3000× *g* for 10 min, and the whey was separated. Syneresis (%) was calculated as a weight of generated supernatant per weight of yogurt multiplied by 100 [31]. Experiments were performed in triplicate and repeated three times.

### 2.7. Determination of Functionality of Yogurt Produced Using Granulated BPF as Premix

#### 2.7.1. LAB Viability

The viability of the LAB was evaluated upon fermentation using the pour plate technique and serial dilutions in phosphate buffer saline (1% PBS). *S. thermophilus* was enumerated using M17 agar (pH 7.2) under aerobic incubation at 37 °C for 48 h. After anaerobic incubation at 37 °C for 72 h, *L. bulgaricus* was counted on MRS agar (pH 6.2). The results were expressed as the log of the mean number of the colony-forming units (log CFU/mL).

#### 2.7.2. In Situ Antibacterial Activity

Cow milk was inoculated with 6 log CFU/mL of *E. coli* ATCC 8739, fermentation with and without BPF premix was conducted, and the obtained yogurts were stabilized for 24 h. After the stabilization, yogurts’ serial decimal dilutions were prepared, plated on Mac Conkey agar, and incubated for 24 h at 37 °C under aerobic conditions. The results were expressed as log CFU/mL.

#### 2.7.3. Cytotoxicity

The cytotoxic effect was estimated using an MTT assay [26]. For the purpose of cytotoxicity testing, yogurt supernatants (i.e., extracted whey) were prepared as previously described [26]. In brief, yogurt samples were centrifuged at 20,000× g for 60 min at 4 °C and filtered using a 0.45 µm syringe filter. The pH was adjusted to 6.8. The filtrates obtained from the control yogurt (without BPF) and yogurt with 9% BPF premix were diluted three times in DMEM. HCT116 cells were treated with diluted supernatants and incubated for 24 h. After an additional incubation (3 h) of the cells with MTT (3-(4,5-dimethylthiazol-2-yl)-2,5-diphenyltetrazolium bromide) solution and dissolution of the formed formazan crystals in DMSO, the viability of the cells was determined by measuring the absorbance at 570 nm using a microplate reader (Multiskan FC, Thermo Scientific, Shanghai, China). Three independent experiments in sextuplicate were performed.

### 2.8. Yogurt Palatability Assessment

#### 2.8.1. Trial with Humans—JAR Test

A consumer test was conducted with 40 untrained panelists (22 females and 18 males, mean age years 40.7 ± 16.4). Fresh yogurt prepared with a 3% BPF premix was evaluated using 5-point just-about-right (JAR) scales (1 = too little, 3 = JAR, and 5 = too much) for the intensity of ‘color’ (too light–JAR–too dark), ‘sourness’ (not sour enough–JAR–too sour), ‘beetroot taste’ (too weak–JAR–too strong), and also, the 5-point hedonic scale (1 = dislike extremely, 3 = neither like nor dislike and 5 = like extremely) for ‘color acceptance’, ‘odor acceptance’,‘ taste acceptance’, ‘acceptance of sourness‘, and ‘texture acceptance’. Consumer acceptance data were subjected to mean drop analysis, as described by Tomić et al. [32]. In addition, three background questions were asked: (1) What is your frequency of consumption (less than once a week or more than once a week) of yogurt? (2) What is your frequency of consumption of beetroot-based products? and (3) Would you share a BPF yogurt with a dog?

#### 2.8.2. Trial with Dogs—The Two-Bowl Test

Eleven healthy, vaccinated, and dewormed adult dogs of varying ages (4 males, 7 females; mean age years 4.28 ± 3.08 years) were included in the present study. Dog breeds and neutered status are presented in Supplementary Table S1. The body condition of all dogs was considered to be ideal (ribs can be palpated through slight fat cover) or moderately overweight (difficult to palpate ribs due to moderate fat cover). Dogs were housed individually in controlled home environments under the supervision of trained staff.

The trial was performed using the two-bowl test [33] administered in the morning for two consecutive days. Yogurt fermented with 3% BPF and commercial yogurt with raspberry were pairwise compared. The ingredients of the commercial dog yogurt are presented in Supplementary Table S2. The amount of provided meal each morning, including tested and regular food, did not exceed the dogs’ recommended total daily intake. Two tested, pre-weighed diets (50 g each) were simultaneously offered to the dogs in two identical bowls. The position of the bowls was changed daily to prevent side bias (left or right bowl preference). The food the dog chose first (first food visited) and the amount of eaten food were noted. Prior to data collection, a two-day adaptation period was provided to familiarize the dogs with the new food and to examine whether dogs display a neophobic behavior and a tendency to side bias. For these purposes, imitating the conditions of the two-bowl test, a common diet that the dogs were accustomed to, and the yogurt fermented with 3% BPF were served. Dogs that exhibited position bias were excluded. Water was offered ad libitum.

The intake ratio of the tested diets was calculated as follows:

(4) $$\mathrm{Intake}\;\mathrm{ratio}\;\mathrm{of}\;\mathrm{BPF}\;\mathrm{yogurt}=\frac{\mathrm{intake}\;\left(g\right)\;\mathrm{of}\;\mathrm{BPF}\;\mathrm{yogurt}}{\mathrm{intake}\;\left(g\right)\;\mathrm{ofBPF}\;\mathrm{yogurt}\;+\;\mathrm{intake}\;\left(g\right)\;\mathrm{of}\;C\;\mathrm{yogurt}}$$

(5) $$\mathrm{Intake}\;\mathrm{ratio}\;\mathrm{of}\;C\;\mathrm{yogurt}=\frac{\mathrm{intake}\;\left(g\right)\;\mathrm{of}\;C\;\mathrm{yogurt}}{\mathrm{intake}\;\left(g\right)\;\mathrm{of}\;\mathrm{BPF}\;\mathrm{yogurt}\;+\;\mathrm{intake}\;\left(g\right)\;\mathrm{of}\;C\;\mathrm{yogurt}}$$

Fresh urine and fecal samples were analyzed before and after the two-bowl tests. The urine collected from each animal by natural voiding was visually inspected for color and described as: light-yellow, yellow, dark-yellow, and pink or red (colored urine sample due to beetroot intake). Fecal samples score was determined as described previously [34].

### 2.9. Surveying of Consumers’ Attitude towards Novel Product and Accompanied Concept

To gain insight into the owners’ attitudes toward yogurt intended for dog nutrition and current feeding practices, a paper questionnaire and an online questionnaire were administrated (Google form). The link to the open survey was shared through a public dog owners’ group on a social media website (Facebook), and the paper questionnaire has been placed at a veterinary clinic (Line Alba, Belgrade, Serbia). Owners completed the paper questionnaire at the clinic at the time of the appointment. One response per participant was allowed. The data collection ran for one month. The questionnaire contained 32 closed questions (all mandatory), about the owner (personal and household data), the dog’s signalment (age, sex, body weight, and health status) and the issues associated with animal’s welfare, owner’s preferences regarding pet food, and the finances allocated for dog-maintenance. The survey participation was anonymous and on a voluntary basis. The survey was created as described by Morelli et al. [35] and is available in translated form as Supplementary material.

### 2.10. Statistical Analysis

The following programs for statistical data analysis were combined and used: software GraphPad Prism 6.0 (GraphPad Software Inc., San Diego, CA, USA) and Excel 2016 (Microsoft). The data obtained from pH, syneresis, bacterial counts, in situ antibacterial activity, and MTT assay were analyzed by analysis of variance (one-way ANOVA, Dunnett’s multiple comparisons test, and Tukey’s honestly significant difference test (HSD)). The level of statistical significance was defined as *p* < 0.05. The data collected from the nutritional questionnaire were transferred into a spreadsheet (Excel, Microsoft) and submitted to descriptive analysis.

## 3. Results and Discussion

### 3.1. Production and Proximate Composition of BPF

The proximate composition of BPF obtained by dehydration and grinding to particle size >200 µm was in line with the previously published results [5]. Carbohydrate content was 34.58 ± 0.23%, whereas the contents of protein and fat were 13.85 ± 0.34% and 0.92 ± 0.12%, respectively. Relatively rich ash portion (8.73 ± 0.21%) can be attributed to high mineral content. A low moisture level (5.85 ± 0.14%) was achieved. A significant amount of crude fiber (36.1 ± 0.47%) confirmed that BPF represents the outstanding source of DF. Owing to the important role in digestibility, laxation, and stool quality, beet pulp has often been used as the primary DF source in dogs’ diets [6].

### 3.2. Granulation of BPF with LAB Using the Fluidized Bed Technique

The BPF produced was granulated successfully with LAB by using fluidized bed, chosen as it consumes less energy for the immobilization of beneficial microorganisms than freeze-drying [15]. In contrast to air drying, fluidized bed is characterized by optimal heat and equal temperature distribution that allows efficient drying of sensitive compounds and microorganisms [15]. The fluidized bed technique was applied to the commercial production of dried baker’s yeast [18], and probiotic encapsulates (e.g., Probiocap™) [36]. However, immobilization of LAB on a minimally processed pomace via fluidized bed has not been conducted to date. To our knowledge, fluidized bed was used until now only as one of the processing steps to obtain carrot pomace powder [37].

### 3.3. Physical and Technological Properties of BPF before and after Granulation

#### 3.3.1. Flowability

The bulk density before and after wet fluidized bed granulation of BPF with 5% *w*/*v* of lactose solution containing LAB was 0.44 g/mL and 0.41 g/mL, respectively, whereas tapped density was 0.55 g/mL and 0.48 g/mL. Based on Carr Index and Hausner Ratio (C_I_ = 21.54, H = 1.27), the flowability of BPF was defined as possible [38]. Significantly improved flowability was ascribed to granulated BPF (C_I_ = 14. 98 and H = 1.18). According to [38], flowability expected for powder characterized by C_I_ = 14.98 and H = 1.18 belongs to Good/Free flow. Classification of powders according to Carr Index and H ratio enable better insight into the improvement of the flowability of initial flour achieved by granulation. Flowability of granulated BPF secures easier manipulation during transport, packaging without dusting, congestion, or spilling.

#### 3.3.2. Particle Size Distribution

**Figure 1:**
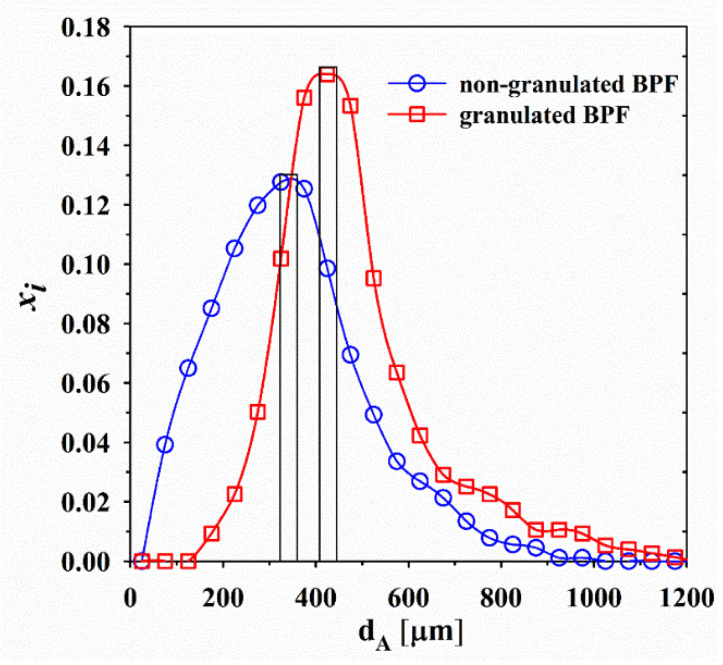
Fractional particle size distribution of BPF before and after wet fluidized bed granulation with 5% *w*/*v* of lactose solution containing LAB at a pilot-scale level.

The comparison of fractional particle size distribution with respect to the projected particle diameter d_A_ for BPF before and after granulation is shown in Figure 1. The particle distribution of non-granulated BPF was in the interval 94 < d_A_ < 916 μm; whereas after the granulation the range was 163 < d_A_ < 1172 μm. The particle size increased by approximately 30%. The particle size enlargement via wet granulation improved the flowability of BPF.

#### 3.3.3. Storability of Non-Granulated and Granulated BPF

Water activity followed weekly, during a month of storage, confirmed superior storability of granulated BPF (Table 1).

### 3.4. Evaluation of Granulated BPF Toxicity

Han: NMRI mice (18–22 g) fed with 50 mg of granulated BPF did not display any signs of toxicity within 72 h of observation. Therefore, BPF could be labeled as safe for consumption.

### 3.5. Application of Granulated BPF as Premix for Yogurt Production

#### Influence of BPF Premix on Yogurt pH and Syneresis

**Figure 2:**
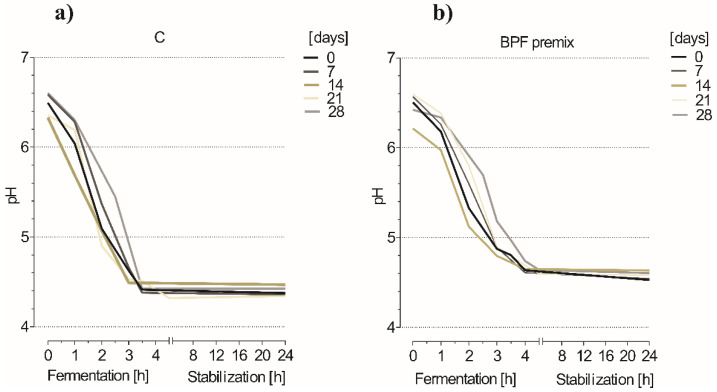
Decrease in pH values during yogurt production (**a**) without (control (C)) and (**b**) with 3% BPF premix. Yogurt samples were prepared weekly with neat starters and the BPF premix stored for up to 28 days (4 °C).

The pH, acidity, and syneresis are important indicators of yogurt quality. Acidity, more precisely the characteristic acidic taste of yogurt, is formed as a result of the presence of lactic acid, diacetyl, and acetaldehyde produced during the fermentation process [21]. Fermentation time required for the preparation of yogurts with neat starter cultures and the BPF premix moderately differed (Figure 2). Time required to reach pH 4.6 with samples stored up to 3 weeks were between 2 h 40 and 3 h 20 min for neat starters and between 3 h 50 and 4 h 30 min for the BPF premix. However, neat and immobilized cultures stored for 4 weeks had slightly slower fermentation. Contrary to our results, beetroot enriched yogurt had lower pH values in comparison to plain yogurt [13]. Yadav et al. [13] reported that the pH of the yogurt samples decreasing upon beetroot powder addition was related to the acidic nature of beetroot. However, the amount of beetroot powder added to achieve such results was two folds higher (6%).

**Figure 3:**
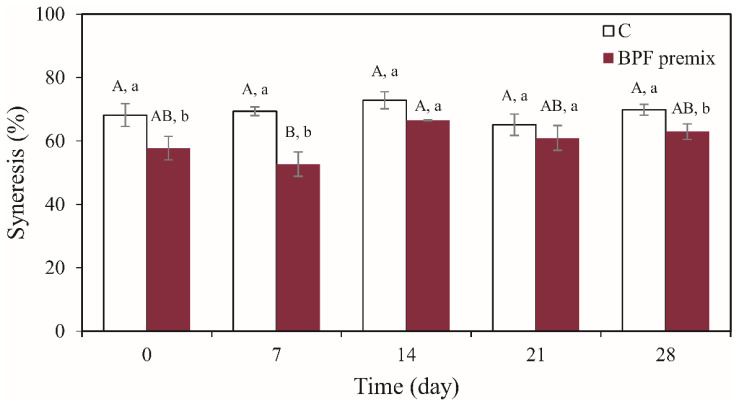
Amount of whey segregated (%) from yogurt prepared by applying neat (control (C)) starter cultures and the 3% BPF premix. Values are presented as mean ± standard error (*n* = 3); ^AB^ a significant difference in means during time within the same sample, ^ab^ a significant difference of means between samples at the same time according to the HSD test (*p* < 0.05).

Higher syneresis rates indicate fermented dairy products’ inferior quality and are attributed to milk preparation, the coagulation process, and the ingredients used [26]. Yogurts produced with the BPF premix had less pronounced syneresis than the plain one (Figure 3). In particular, yogurts produced at the beginning of the starter’s storage (upon 0 and 7 days) exhibited such a property. The lower percent of syneresis was already ascribed to the yogurts supplemented with immobilized cells rather than to those receiving free ones [14]. Fruit and vegetable segments, as well as vegetable powder, can reduce syneresis owing to their hygroscopic nature [14]. Accordingly, the high water-holding capacity of beetroot pomace could explain lower syneresis rates.

### 3.6. Functionality of Yogurt Prepared Using Granulated BPF as a Premix

#### 3.6.1. Bacterial Viability

The change in the microbial counts was monitored in fresh yogurts prepared weekly for one month with the stored BPF premix and neat starters. In accordance with previous findings [14,21], the viability of *S. thermophilus* was higher than that of *L. bulgaricus* (Table 2). Different oxygen tolerance of strains caused the survival rate distinction. The ability to neutralize reactive oxygen species (ROS) depends on enzymatic equipment. The low capacity of *L. bulgaricus* to eliminate H_2_O_2_, or other ROSs could be related to poor equipment with antioxidative enzymes, such as catalase, peroxidase, and superoxide dismutase. Paradoxically, under the microaerobic conditions, LAB, including *L. bulgaricus*, produced H_2_O_2_, which resulted in growth arrest. LAB is often exposed to oxygen during fermentation, product handling, and storage [39]. Importantly, neat and immobilized starters were stored in oxygen permeable bags. Thus, the oxygen exposure might have negatively affected the growth of *L. bulgaricus*, with a less significant impact on the viable count of *S. thermophilus* [21].

In line with [14], the high viability of *S. thermophilus* was maintained upon immobilization and after the storage (CFU > log 8.5 after 4 weeks of storage). Moreover, *L. bulgaricus* counts were higher, by 18.34%, when a 4-week stored BPF premix (log 5.67) was used instead of neat starters (log 4.83). The result could be considered to be a promising initial step to overcoming the challenge of sustaining the high viability of *L. bulgaricus*, especially for a longer period of storage. However, the link between the length of LAB storage and their reduced survival rates was evident. Similar to our findings, *L. plantarum* cells fluidized with glucose, trehalose, sucrose, and maltodextrin as protectants and lost their initial viability after one month of storage [15].

These results confirmed that BPF could be used as matrices for the immobilization of yogurt starter cultures. Presumably, BPF enabled the natural entrapment of LAB, providing physical adsorption by electrostatic forces and covalent binding between the cell wall and the carrier [14]. However, higher viability of LAB might be achieved by an additional coating of BPF granulate. Alginate, chitosan, or lipid-based coating already suggested for that purpose [36,40,41] remained to be used in the following phase of the investigation.

#### 3.6.2. In Situ Anti-Escherichia Activity

*E. coli* is often presented as a foodborne gram-negative bacteria that can contaminate milk-based products [42]. Here, the viability of *E. coli* in yogurts fermented with neat starters and the BPF premix was investigated, and anti-*Escherichia* properties were detected in all samples (Table 3). The yogurts fermented with neat starters and the BPF premix stored for up to 14 days had the most evident response. Reduction in *E. coli* numbers may be related to the large initial LAB counts required to produce adverse effects on the pathogen [43]. LAB starter culture could exert an antagonistic influence against bacterial pathogens [42]. The LAB metabolic activity responsible for pH dropping and liberating potent antibacterial peptides from milk proteins could be a limiting factor for the growth of *E. coli* [22,42]. Supernatants of plain yogurt and yogurt supplemented with pineapple peel powder also inhibited the growth of *E. coli* [22].

BP usage as a functional ingredient with antibacterial activity was suggested based on the finding that a high concentration of BP extract (100 mg/mL) inhibited *E. coli* viability [43]. A slight increase in the antimicrobial activity of BPF yogurt observed here can be related to the low share of BPF (3%). It might be supposed that a higher portion of BPF would further increase the antimicrobial properties.

#### 3.6.3. Cytotoxic Properties

**Figure 4:**
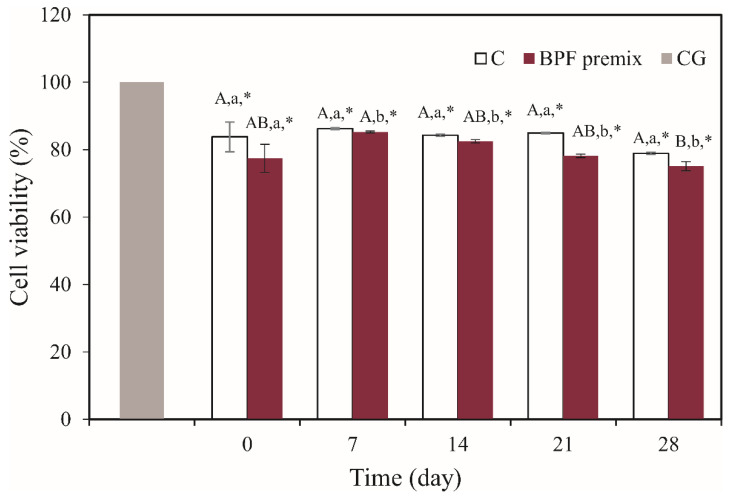
Inhibition rates of HCT116 cells treated for 24 h with supernatants from yogurts prepared by applying neat starter cultures (control (C)) and BPF premix. CG; cell growth control. Values are presented as mean ± standard error (*n* = 3); ^AB^ a significant difference in means during time within the same sample; ^ab^ a significant difference in means between samples at the same time according to HSD test (*p* < 0.05); * a significant difference in means between all samples and CG according to the Dunnett’s test (*p* < 0.05).

The cytotoxic potential of the supernatants of yogurts prepared with neat starters and the BPF premix were tested on the human colon cancer cell line HCT116. For all tested samples, a statistically significant reduction of cell viability was observed (13.02–24.47%) (Figure 4). However, cells were more susceptible to supernatants obtained from BPF yogurts. The cytotoxic effect of the yogurt supernatants on HCT116 cells was established earlier [26]. Supernatants of yogurts fermented with apple pomace [26] and pineapple peel powder [22] exhibited more pronounced inhibition of colon cancer cell viability (HCT116 and HT29, respectively) than plain yogurt supernatants. Several bioactive peptides liberated from milk proteins exhibit cytotoxic activity against malignant cells by disrupting cell membranes, modulating the cell cycle, and promoting apoptosis [22]. Betalains were also found responsible for potent anticancer activities [7,11]. A few in vivo studies confirmed that LAB exerts anticarcinogenic effects and that diet-induced microfloral alteration may prevent the development of colon cancer [44].

### 3.7. Palatability Study

#### 3.7.1. Trial with Human Panelists

Data reported in the literature related to the sensory analysis of beet dairy beverages are quite diverse. Smoothies with beets had high acceptability in terms of color, but the intense earthy flavor was not described as pleasant [12]. However, yogurt enriched with beetroot powder gained higher acceptance for color, texture, flavor, and overall acceptability [13]. In accordance with these findings, average hedonic scores for ‘‘color acceptance’’, ‘‘odor acceptance’’, ‘‘taste acceptance’’, ‘’acceptance of sourness’‘, and ‘’texture acceptance’’ of BPF yogurt were 4.68, 4.05, 3.43, 3.30, 3.50, respectively, i.e., more or less within the range of ‘’like a little’’ category. Thus, the majority of tested respondents liked the BPF yogurt.

**Figure 5:**
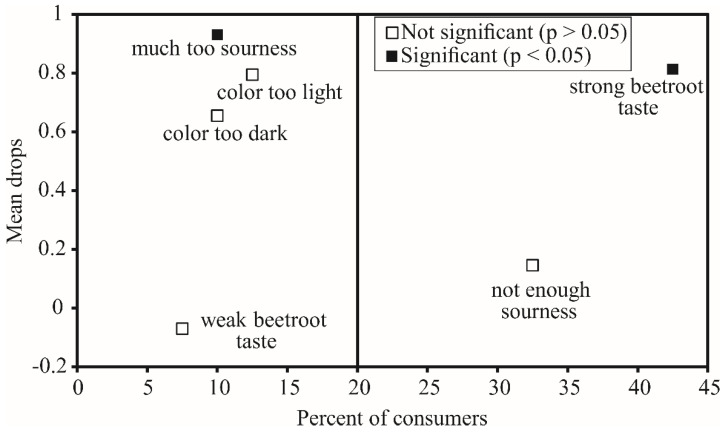
Mean drop analysis of the BPF yogurt sample (*N* = 40).

The results of the mean drop analysis are shown in Figure 5. Statistically significant mean drops (*p* < 0.05) in sufficiently large consumer groups (≥20%) were noted only for beetroot taste (42.5%). The ‘‘beetroot taste’’ could be softened by using pomace flour obtained from a different selection of beetroot varieties and by adding other kinds of vegetable or fruit pomace flour. Further, adding probiotic strains could modify the taste and contribute to masking the taste of beets.

#### 3.7.2. Feeding Trial with Dogs

The yogurt prepared with BPF premixes was chosen first more often than commercial yogurt with raspberry (13:5). “First bite” is commonly assumed to be related to the aromatic characteristics of the food, and thus it is a critical feature in palatability assessment [33]. Eight out of ten dogs showed no resistance to eating; there were no leftovers from tested foods. On the second day of testing, two dogs tasted neither BPF yogurt nor the control product. Unlike other canines’ diet that is based on dry food, their meals mostly include raw or boiled meat. Total food intake was 0.45 for both products. Other important criteria for pet foods’ evaluation are stool consistency and quality. The consumption of BPF yogurt did not affect fecal consistency. Stool quality was rated with values 3 or 4 according to the Bristol stool scale. Further, none of the urine samples appeared colored (pink or red) due to BPF yogurt intake.

### 3.8. Consumers Attitude Survey

As a potential link between the basal canine diet and their preferences for functional beverages was detected, a questionnaire related to the owners’ attitudes towards functional treats, particularly yogurt intended for dog nutrition and current dogs’ feeding practices, was administrated. A total of one hundred dog owners participated in this survey. The owner’s data and canine signalment are presented in Supplementary Table S3. Importantly, most dogs (90%) had an ideal body condition according to their owner’s estimation. Furthermore, overweight condition and obesity are the most underrated common disorders. According to the data reported in the literature [45], the body conditions of the dogs are probably underestimated when they are based on the subjective assessments of the owners.

A significant percent of the survey respondents are aware that dogs could consume yogurt (67%) and that fermented dairy products may have beneficial effects (49%) on canine health. However, only 17% of dog owners are familiar with the fact that yogurts designed for dog nutrition exist on the market. Moreover, only 20% of dog owners were informed that dogs are allowed to consume beets. Therefore, it is important that information considering the health benefits of functional, fermented dairy products for dog feed becomes widely available to owners. Above all, it is crucial that owners become aware of the actual body condition of their companion animals. Moreover, the biggest financial burden when maintaining a dog for most people is related to pet healthcare costs (43%). A healthy dog diet can prevent the development of various diseases. Food containing fibers could increase satiety, decrease blood cholesterol concentrations, and promote gut commensal bacteria growth [6]. Our previous study demonstrated that the addition of apple pomace into high fat and sugar, as well as standard diet, decreased feed efficiency ratio significantly and improved glucose tolerance [46]. Thus, yogurts with BPF premix should be considered as adequate functional treats and included in an appropriate feeding plan. Furthermore, the owners are largely responsible for the obesity of their companion animals. Moreover, humans and canines share most of the causative factors and the mental/physical epidemiology associated with overweight problems [47]. Therefore, the concept of sharing a fiber-enriched meal between dogs and owners is imposed. According to our results, most of the respondents (80%) who participated in the palatability study/trial with a human panelist stated that they would like to share BPF yogurt with a dog.

## 4. Conclusions

An innovative way of beetroot pomace utilization in human and animal nutrition was demonstrated. As a source of DF and LAB carrier, BPF was successfully employed to obtain a fermented beverage with improved functional properties. Wet fluidized bed was estimated as a feasible technique to achieve better physical properties and storability of BPF and to employ it as adequate support of LAB starter culture. The applicability of granulated BPF conceptualized as a novel DIY formula for obtaining a fresh, nutritious yogurt suitable for human and dog consumption was confirmed. To our knowledge, this is the first study that focuses on the application of pomace granulated with LAB as a premix for fermented beverages. The core concepts of BPF premix were the functionality and palatability of yogurts amended to both humans and dogs. Enrichment of BPF yogurt with 1% DF *w*/*w* improved cytotoxic activity against the human colon cancer cell line and anti-*Escherichia* activity, leading to the conclusion that its introduction into the diet might contribute to the well-being of consumers and their animal companions. Acceptable sensory properties for both humans and dogs, and positive consumers’ attitude toward granulated BPF as a premix, provided useful guidance and initial evaluation of its market prospective. The concept of sharing food with pets was also positively received by consumers. Additional improvement of yogurt functionality and palatability, as well as LAB viability by the further coating of granulated BPF with various protectants, remains to be investigated in the near future.

## 5. Patents

Gorjanović, S., Zlatanović, S., Jovanović, M., Đurišić, M., Micić, D., Šoštarić, T., Lopičić Z. Granulates of fruit and vegetable flours with prebiotics and lactic acid bacteria P-2021/0695, 2021. Patent Application.

## Figures and Tables

**Table 1:** Water activity of non-granulated and granulated BPF during a month of storage at 4 °C.

Water Activity of (aw)		
Day	Non-Granulated BPF (10 g)	Granulated BPF (10 g)
0	0.311 ± 0.004 ^D,a^	0.208 ± 0.001 ^E,b^
7	0.317 ± 0.001 ^CD,a^	0.239 ± 0.002 ^D,b^
14	0.322 ± 0.001 ^C,a^	0.259 ± 0.001 ^C,b^
21	0.333 ± 0.001 ^B,a^	0.279 ± 0.001 ^B,b^
28	0.343 ± 0.001 ^A,a^	0.290 ± 0.002 ^A,b^

BPF samples were stored for up to 28 days (4 °C). Values are presented as mean ± standard error (*n* = 3); different uppercase superscript in the same column indicates a significant difference in means during time, and different lowercase superscript within the same row indicates a significant difference in means at the same time, according to HSD test (*p* < 0.05).

**Table 2:** Viable counts (log CFU/mL) of *S. thermophilus* and *L. bulgaricus* in yogurts prepared by applying starter cultures (control) and granulated BPF as a premix.

LAB	*L. bulgaricus*		*S. thermophilus*	
S *	7.54 ± 0.05		10.18 ± 0.15	
Day	Control	BPF	Control	BPF
0	7.07 ± 0.11 ^A,a^	7.00 ± 0.29 ^A,a^	9.21 ± 0.11 ^A,a^	9.10 ± 0.01 ^AB,a^
7	6.82 ± 0.10 ^AB,a^	6.11 ± 0.30 ^AB,a^	9.15 ± 0.13 ^AB,a^	9.19 ± 0.08 ^A,a^
14	6.37 ± 0.29 ^AB,a^	6.26 ± 0.44 ^AB,a^	8.99 ± 0.12 ^AB,a^	9.13 ± 0.12 ^AB,a^
21	5.85 ± 0.18 ^BC,a^	5.77 ± 0.18 ^B,a^	8.70 ± 0.22 ^B,a^	8.68 ± 0.16 ^BC,a^
28	4.83 ± 0.24 ^C,a^	5.67 ± 0.23 ^B,a^	8.87 ± 0.02 ^AB,a^	8.51 ± 0.17 ^C,a^

* S-initial counts (log CFU/mL) of strains in solution before fluidized bed drying; values are presented as mean ± standard error (*n* = 3), different uppercase superscript in the same column indicates a significant difference of means during that time; different lowercase superscript within the same row indicates a significant difference in means at the same time, according to the HSD test (*p* < 0.05).

**Table 3:** In situ anti-*Escherichia* activity of yogurt samples fermented with neat starters and granulated BPF as a premix in comparison to growth control (GC).

Days of BPF Storage	Viable Counts of *E. coli* (log CFU/mL)	
	Yogurt with Neat Starters	Yogurt with BPF
0	5.96 ± 0.02 ^AB,a,^*	5.76 ± 0.12 ^A,a,^*
7	5.57 ± 0.14 ^B,a,^*	5.26 ± 0.21 ^A,a,^*
14	5.48 ± 0.07 ^B,a,^*	5.81 ± 0.16 ^A,a,^*
21	6.74 ± 0.25 ^A,a^	6.67 ± 0.38 ^A,a^
28	6.74 ± 0.17 ^A,a^	6.39 ± 0.47 ^A,a^
**GC**	6.85 ± 0.14	

Values are presented as mean ± standard error (*n* = 3); ^AB^ a significant difference in means between samples in the same column; ^ab^ a significant difference in means between samples in the same row according to HSD test (*p* < 0.05); *significant difference between samples and GC (growth control) according to the Dunnett’s test (*p* < 0.05).

## Data Availability

The data presented in this study are available on request from the corresponding author.

## Supplementary Materials

The following are available online at https://www.mdpi.com/article/10.3390/foods10081696/s1, Table S1: The dog’s signalment, Table S2: Ingredients of the commercial yogurt, Table S3: Nutritional survey. Survey respondents’ demographics (A), dogs signalment (*N* = 100) (B), financial requirements (C), and dog feeding practices (D), Material S Survey.
