# Supplementary material for: Functionality and Palatability of Yogurt Produced Using Beetroot Pomace Flour Granulated with Lactic Acid Bacteria
Source: Foods. 2021 Jul 22;10(8):1696. doi: 10.3390/foods10081696 (PMC8392337; doi:10.3390/foods10081696)
Supplement: Supplementary file 1 [file foods-10-01696-s001.zip › foods-1285098.pdf]

Supplementary

Table S1. The dog's signalment

Breed	The total number	Neutered status
Miniature Schnauzer	1	spayed female
Miniature Poodles	1	sexually intact male
Maltese*	1	spayed female
Mongrel	4	sexually intact male
		castrated male
		spayed female
		spayed female
Belgian Malinois		spayed female
Siberian Husky	2	spayed female
		sexually intact male
Staffordshire Bull Terrier	1	spayed female

* A dog that exhibited position bias was excluded from the study.

Table S2. Ingredients of the commercial yogurt

Compound	Amount [%]
Yogurt	78
Raspberry	16
Agave syrup	6

Table S3. Nutritional survey. Survey respondents demographics A), dogs signalment (N=100) B), financial requirements C), and dog feeding practices D).

**A) Dog owners**	N (%)
**Gender**	
Male	19
Female	81
**Age**	
< 25	51
25–40	30
40–60	16
> 60	3
**Household**	
Single	15
Couple	19
Family	28
Multi-member community	38

**C) Financial requirements**	N (%)
**Dog maintenance costs per month (EUR)**	
< 50	34
50–100	53
100–200	11
>2 00	2
**The most common source of cost**	
Dog treats	16
Dog health care	43
Accommodation for dogs	3
Dog grooming salon	14
Dog toys	9
Training equipment	4
Other	11

**B) Dogs**	N (%)
**Gender**	
Male	46
Female	54
**Neutering status**	
Neutered	45
Not neutered	55
**Age**	
< 1	11
1–2	23
2–7	45
7–12	15
> 12	6
**Body condition**	
Underweight	-
Normal weight	90
Overweight	10
**Weight, kg**	
< 8	29
8–12	12
12–15	15
15–20	10
20–25	12
25–30	9
30–35	6
35–45	3
> 45	4
**Breed's size**	
Small-size dogs	36
Medium-size dogs	50
Large-size dogs	14

**D) Claim**	**Viewpoint** N (%)		
	Yes	No	I don't know
Dogs are allowed to drink yogurt	67	29	4
Dogs are allowed to eat beetroot	20	9	71
A dog's diet determines its health	95	2	3

Material S Survey

Owner data

1. Sex of the dog owner
    - Male
    - Female
2. Age of the dog owner
    - < 25
    - 25-40
    - 40-60
    - >60
3. How often do you take the dog to the veterinarian?
    - Only for vaccination
    - When it is ill
    - For vaccination and when it is ill
    - For preventive care, to check for infectious diseases or routine examinations
    - For vaccination, when it is ill, and for preventive care
    - Rarely
4. The dog lives with:
    - one owner
    - two owners (couple)
    - owner / owners with children
    - a multi-member community of people
5. The dog shares living space with:
    - other dog / dogs
    - other pets
    - other
6. The dog's diet mostly includes:

- fresh meat
- granules (dry food) intended for dog nutrition
- soft foods intended for dog nutrition
- food left over from people's meals
- heat-treated vegetables and meat prepared exclusively for pets
- equally includes soft foods and granules
- other

7. The dog lives in:
    - premises of the apartment / house
    - garden of the house
    - other
8. The physical activity of the dog is mostly based on:
    - a walk
    - playing with other dogs
    - running out into the garden
    - sports activities for dogs (swimming, agility)
    - sports activities that the owner and the dog do together (running, hunting, and hiking)
    - other
9. In your opinion, your dog:
    - is malnourished
    - has appropriate body weight
    - is obese
10. How old is your dog (years)?
    - < 1
    - 1–2
    - 2–7
    - 7–12

- > 12

11. Your dog's body weight is:
    - < 8 kg
    - 8–12 kg
    - 12–15 kg
    - 15–20 kg
    - 20–25 kg
    - 25–30 kg
    - 30–35 kg
    - 35–45 kg
    - > 45 kg
12. Your dog belongs to the group:
    - small dog breed
    - medium dog breed
    - large dog breed
13. Your dog is:
    - spayed female
    - castrated male
    - a sexually intact female
    - a sexually intact male
14. I learned what a dog should eat:
    - through a conversation with a veterinarian
    - through a conversation with a dog breeder
    - by searching the internet
    - by reading the professional literature
    - by talking to friends / family who have had dogs for a long time
    - all of the above

15. My dog's diet includes:
    - vegetables
    - fruit
    - meat
    - different plants
    - cereals
    - equally represented all of the above
    - I am taking the recommended food and I have not paid attention to the ingredients
16. Dog food must not contain sugars:
    - I agree
    - I do not agree
    - I am not sure
17. My dog sometimes refuses to eat granules (dry dog food):
    - Yes
    - No
    - I am not sure
18. My dog often refuses to eat granules (dry dog food):
    - Yes
    - No
    - I am not sure
19. The dog's diet determines his health:
    - Yes
    - No
    - I am not sure
20. Do you think that sweets in a dog's diet can have negative effects on his health?
    - Yes
    - No

- I am not sure

21. Do you know there are treats on the market intended exclusively for dogs?
    - Yes
    - No
22. In addition to the standard food, I also give my dog
    - dog treats (biscuits, beef veins, and dried meat)
    - dog yogurt
    - yogurt for human consumption
    - dog ice cream
    - chewing gum for dental hygiene
    - dog chocolate
    - none of the above
23. I give the dog treats that:
    - he likes the most
    - improve his immunity
    - have a beneficial effect on the hair appearance
    - have a beneficial effect on dental health
    - have a beneficial effect on the digestive system
    - have some other health benefits
    - none of the above
24. How often do you give your dog treats?
    - On a daily basis
    - 2 to 3 times a week
    - Once a week
    - 2 to 3 times a month
    - Once a month
    - None of the above

25. Did you know that there are yogurts for dogs on the market?
    - Yes
    - No
26. Fermented dairy products have different effects on dog health?
    - I disagree
    - I agree
    - I am not sure
27. Dogs can drink yogurt:
    - Yes
    - No
    - I am not sure
28. Dogs can eat beetroot:
    - Yes
    - No
    - I am not sure
29. My dog has a digestive disorder:
    - Yes
    - No
30. With commercially available food and treats for dogs, I pay attention to:
    - whether and what additives there are
    - whether and how much sugar there is
    - I am taking the recommended food and I have not paid attention to what is in it
31. On average, I spend on a dog per month (EUR):
    - < 50
    - 50–100
    - 100–200
    - > 200

32. On an annual basis (in addition to the cost of basic food), I spend the most on:

- dog treats
- dog health care
- dog boarding houses
- dog salons
- dog toys
- dog walking and training equipment
- none of the above
